# Atlanta Medical College—Preparatory Course

**Published:** 1872-03

**Authors:** J. G. Westmoreland

**Affiliations:** Dean


### ATLANTA MEDICAL COLLEGE—PREPARATORY COURSE.

This Session of the College will open on the first Monday in May next, under the direction of the Faculty, and continue until the last of August. Attendance will not count as a course in the requisites for graduation, but will afford the student great advantages in becoming thoroughly instructed in the elementary principles of the Medical Sciences, and their practical application, so as to give the beginner an appreciation of Lectures afterwards, and prepare the advanced student for examination at the end of the succeeding regular Winter Course, and for the ultimate duties of professional life.

From the foregoing announcement, it will be seen that a radical change has been made in the time of holding the regular Course of Lectures, and in the mode of teaching, in this Institution. The scholastic year is divided into two terms: one commencing the first of May and terminating the last of August—the other, including the winter months, to continue from October till March.

The Summer Course will consist of Lectures, Recitations, and Practical Exercises, conducted by the Faculty and a full corps of adjuncts to the different Chairs. The exercises will be so arranged as to give all possible advantages to both beginner and advanced student. Instruction and examinations on the fundamental branches of Medical Science, by the adjuncts and Faculty, will be well adapted to the wants of those commencing the study. At the same time, a prominent feature of the Course will be practical teaching and clinical illustrations, consisting of auscultation and percussion; analysis of the urine and other fluids; microscopic examinations of pathological and normal specimens; investigations of diseases of the eye and ear, with the ophthalmoscope and otoscope; and diseases of the throat, nose and larynx, in the study of which the laryngoscope and all other modern appliances will be brought into requisition.

Clinical material is abundant for all these purposes, and the advanced student is expected to engage in such investigations, which will continue through the entire scholastic year, including the two Courses.

The Trustees and Faculty of the College think that, in the successful operation of this plan, one step is made toward a more perfect system of Medical Education. The beginner is often confused by the large number of unconnected facts presented in one day, during a regular Course, and the Preparatory Session, like grammar schools of literary institutions, prepares him for the regular Collegiate Course.

A fee of Twenty Dollars will be required for attendance on the Summer Session, which will be deducted from the price of Tickets for a full Course of regular Lectures during the following Winter.

The Annual Announcement of the regular Course of Lectures, which opens in October, will be issued in due time, and will contain Catalogues of Trustees, Faculty, and all Graduates of the Institution from its organization.

J. G. WESTMORELAND, M.D., Dean.

